# Determinants of health literacy and health behavior regarding infectious respiratory diseases: a pathway model

**DOI:** 10.1186/1471-2458-13-261

**Published:** 2013-03-22

**Authors:** Xinying Sun, Yuhui Shi, Qingqi Zeng, Yanling Wang, Weijing Du, Nanfang Wei, Ruiqian Xie, Chun Chang

**Affiliations:** 1 Department of Social Medicine and Health Education, School of Public Health, Peking University, 38 Xueyuan Road, Haidian District, Beijing, 100191, P.R. China; 2 Department of Program Management, Chinese Center of Health Education, Building 12, Block 1, Anhua Xili, Andingmenwai, Beijing, 100011, P.R. China

**Keywords:** Health literacy, Health behavior, Determinants, Causal pathways

## Abstract

**Background:**

Health literacy has been defined as the degree to which individuals have the capacity to obtain, process, and understand the basic health information and services needed to make appropriate health decisions. Currently, few studies have validated the causal pathways of determinants of health literacy through the use of statistical modeling. The purpose of the present study was to develop and validate a health literacy model at an individual level that could best explain the determinants of health literacy and the associations between health literacy and health behaviors even health status.

**Methods:**

Skill-based health literacy test and a self-administrated questionnaire survey were conducted among 3222 Chinese adult residents. Path analysis was applied to validate the model.

**Results:**

The model explained 38.6% of variance for health literacy, 11.7% for health behavior and 2.3% for health status: (GFI = 0.9990; RMR = 0.0521; χ^2^ = 10.2151, *P* = 0.1159). Education has positive and direct effect on prior knowledge (β = 0.324) and health literacy (β = 0.346). Health literacy is also affected by prior knowledge (β = 0.245) and age (β = -0.361). Health literacy is a direct influencing factor of health behavior (β = 0.101). The most important factor of health status is age (β = 0.107). Health behavior and health status have a positive interaction effect.

**Conclusion:**

This model explains the determinants of health literacy and the associations between health literacy and health behaviors well. It could be applied to develop intervention strategies to increase individual health literacy, and then to promote health behavior and health status.

## Background

Health literacy has been defined as the degree to which individuals have the capacity to obtain, process, and understand the basic health information and services needed to make appropriate health decisions [1,2].Over the last decade, health literacy has become a hot spot of research[3,4]. With a deeper understanding of health literacy in academic circles, more and more researchers find that a lack of health literacy can cause some adverse effects for individuals and society. Low literacy is associated with a variety of adverse health outcomes, including increased mortality, hospitalization, and in some cases poorer control of chronic health conditions [5-9]. Additionally, limited health literacy impacts on the prevention and screening of diseases, health behavior, the taking of patients’ history and the interpretation of diagnoses [10-14]. Knowing little about preventive care, people with low health literacy tend to use more medicines and more expensive healthcare services, including hospitalization and emergency services [7,15,16].

Some investigators have elucidated explained the relationship of between limited health literacy and socioeconomic indicators, health behaviors, and health outcomes [17,18]. Researchers have focused on explaining the potential mechanisms between these variables. Aging, the language barrier, low education, bad socio-economic status and suffering from chronic diseases were all regarded as risk factors of limited health literacy [6,19].

Though limited health literacy has been shown to be associated with worse health outcomes and some socioeconomic characteristics, the causal pathways are not entirely known. Several researches have focused on explaining potential mechanisms. The conceptual model by Baker illustrates these hypothesized relationships by highlighting individual capacities that are associated with literacy skill, the complexity of both printed and spoken health information and other factors such as cultural norms that are relevant to health outcomes [20].

In 2007, Paasche-Orlow and Wolf proposed a conceptual causal model to explain associations between limited health literacy and health outcomes [21]. In their model, socioeconomic indicators are the basic factors influencing health literacy. These include level of education reached, ethnicity, age, occupation and income. Their model distinguishes three different types of health action that mediate the impact of health literacy on health: access to and utilization of health care, patient-provider interaction, and self-care. Each of these domains is defined not only by patient factors but also by external factors that can be attributed to the health care provider or the health system. The pathways are particularly useful in highlighting the role of health actions and providing a useful taxonomy of behavioral domains.

von Wagner’s review introduced a framework drawing on ideas from health psychology and proposing that associations between health literacy and health outcomes could be mediated by a range of health actions involving access to and use of health care, patient–provider interactions, and the management of health and illness[22]. The framework outlines ways in which health literacy might affect either health actions themselves or their motivational and volitional determinants, which have been identified in social cognition models.

McCormack established a conceptual framework for individual health literacy[23]. The framework illustrates how health literacy functions at the level of the individual, while acknowledging that factors external to the individual (including family, setting, community, culture and media) influence all the relationships represented in the model. The framework is organized into four primary elements: (1) health-related stimulus; (2) factors that influence the development and use of health literacy skills, including socio-demographic characteristics, resources , prior knowledge and capabilities; (3) health literacy skills needed to comprehend the stimulus and perform the task; and (4) mediators between health literacy and health outcomes including motivation, attitudes, emotions, and self-efficacy. The health related outcomes include behaviors and status.

Although all these models or frameworks have given the relationship between socio-demographic characteristics, prior knowledge, health literacy, health behavior/action and health outcomes, they are all theoretical explanations. Few studies have tried to validate them through the use of statistical modeling. So this study aimed to develop a health literacy model and to statistically validate it using path analysis.

### Hypothesis – a health literacy model

**Figure 1:**
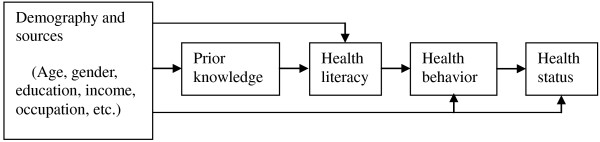
Hypothesis of a health literacy model at an individual level.

With the models of Baker, Paasche-Orlow, von Wagner and McCormack for reference, we proposed a health literacy model at an individual level. Figure 1 represents this model. In this model, socio-demographic indicators, including age, gender, level of education reached, occupation and income, are the basic factors influencing other variables. Besides socio-demographic indicators, prior knowledge also influences the development of health literacy skills. Then health literacy has direct effect on health behavior, meanwhile, as a mediator between prior knowledge and health behavior. Finally, health behavior influences health status.

## Methods

### Questionnaire

The first part of the questionnaire was concerned with socio-demographic characteristics including age, gender, ethnicity, household registration status, marital status, education, occupation and income. The second part measured knowledge of infectious respiratory diseases, known as prior knowledge. Questions were asked about the different types of infectious respiratory diseases and their prevention methods. The maximum possible score for this part of the questionnaire was 12. The third category asked about individual behaviors and actions including washing hands, wearing a face mask, sneezing, room ventilation and treatments for infectious respiratory diseases. The maximum possible score for the health behaviors category was 23. The last part of the questionnaire was concerned with individual health status. Information sought included how frequently the subject fell sick, how often they saw a doctor, the degree of severity for each sickness as well as the duration of the sickness. This category was marked with a maximum score of 13.

### Skill-based health literacy instrument

A skill-based health literacy instrument was established using Ratzan and Parker’s (2000) definition of health literacy: “The degree to which individuals can obtain, process, understand, and communicate about health-related information needed to make informed health decisions [1].The instrument included sixteen stimuli materials involving the distribution of epidemics, immunization programs, early symptoms, means of disease prevention and individual’s preventative behavior. The instrument included five different subscales: print-prose, print-document, print-quantitative, oral and internet. The print-prose scale measured the knowledge and skills needed to search, comprehend, and use information from texts that were organized in sentences or paragraphs, while the print-document scale measured from non-continuous texts in various formats. The print-quantitative scale measured the knowledge and skills needed to identify and perform computations using numbers embedded in printed materials [24].

McCormack developed a more comprehensive measure of health literacy, named the Health Literacy Skills Instrument (HLSI). Similar to other studies, this instrument measures print literacy. However, it was innovative in that it also uses non-print stimuli and examines oral and internet-based information seeking skills [25]. In this study, oral literacy was tested though six questions from three pieces of audio or video. We too used non-print stimuli and measured oral and internet-based skills, but we did so using a series of questions to test the ability of internet-based information seeking rather than having the participants actually seek information online.

The measurement instrument consisted of 30 items (Table 1): five concerning print-prose literacy, eight for print-document literacy, six for print-quantitative literacy, six for oral literacy and five for internet-based information seeking literacy. The overall degree of difficulty and discrimination of the instrument were 0.693 and 0.482 respectively. The instrument demonstrated good internal consistency reliability with a Cronbach’s alpha of 0.864. As for validity, confirmatory factor analysis showed that the items were grouped into five subscales representing prose, document, quantitative, oral and internet based information seeking skills. While the first three instruments pertained to print health literacy, the last two pertained to multimedia health literacy (χ2 = 9.200, P > 0.05, GFI = 0.998, TLI = 0.988, AGFI = 0.992, RMSEA = 0.028).

**Table 1:** Reliability and construct validity of the instrument on health literacy regarding infectious respiratory diseases

**Varibles**	**Items**	**Range**	**Item example**	**Cronbach α**
Prior knowledge	12	0-12	Is tuberculosis a kind of infectious respiratory disease?	0.662
Health behavior	23	0-23	How should you do when coughing or sneezing?	0.688
Health status	13	0-13	How many times did you go to see a doctor due to catching a cold in the last six months?	0.623
Health literacy	30	0-30		0.863
Print-prose	5	0-5	Stimuli: A poster describes flu-preventive behaviors.	0.568
			Question: Which behavior is not helpful for flu prevention?	
Print-document	8	0-8	Stimuli: A map describes the number of local cases of Influenza A (H1N1), as well as the national total, for different provinces all over China at 11th June 2009.	0.664
			Question: How many local infected cases of Influenza A (H1N1) appear in Beijing?	
Print-quantitative	6	0-6	Stimuli: A table describes the expense account submitting system of the New Rural Cooperative Medical System in China.	0.531
			Question: A man was in his county’s hospital for his asthma. Besides the self-paid part, the total expense is 2200 Yuan. How much money can he apply for reimbursement from NCMS office for?	
Oral	6	0-6	Stimuli: The video describes early symptoms of tuberculosis and some policies concerning the disease.	0.624
			Question: Where can you get free diagnosis and treatment for TB according to public policy?	
Internet	5	0-5	Can you search for some information about “measles vaccine immunization” using the internet?	0.964

### Target population and sampling

Between May and December 2011, surveys were carried out in Beijing city (The capital of China), Datong city (in Shanxi province, North China) and Shenzhen city (in Guangdong province, South China). Multi-stage sampling was employed. The target population was first stratified into residents from cities and residents from villages (living in cities at the time and having lived for more than 6 months), with an equally divided sample size. They must be more than 16 years old. Then, based on the principle of balancing samples among factors like age and occupation, cluster sampling was conducted in six places where locals gather (including communities, factories, government organizations and other institutions), and six places where non-local residents gather (including hotels, building sites, assembly shops and employment medical examination centers).

The sample size was calculated by the function *n* = *Z*^*2*^_*1-a/2*_*P* (1 - *P*)/*d*^2^ × deff. According to data obtained from the National Health Literacy Survey in 2008 regarding health literacy towards infectious diseases, the expected percentage was 16% (P = 0.16) [26]. The minimum sample size is 2581. Considering recovery rates and efficiency rates of the questionnaire, the actual sample size should be at least 3186. In total, 3222 residents responded to the survey.

### Procedures

The study received approval from the Peking University Institutional Review Board and the approval number is IRB00001052-10101. It was also accordance with Helsinki Declaration. The investigations were performed in large multimedia conference rooms. The survey was carried out by trained investigators. Information about the study was provided by the investigators and informed consent was obtained from each participant. All participants were then instructed to answer the questions that related to audio & video materials. The rest of the questionnaire was answered by participants themselves.

### Statistical analysis

In order to ensure the quality of data, questionnaires with more than 10% of items unanswered were considered ineligible and removed before analysis. EPI Data 3.0 was used for data double entry and SPSS 13.0 for data analysis. Descriptive statistics were employed to examine demographic characteristics. ANOVA was applied to compare the differences among social demographic groups and the Student-Newman-Keuls method was used to control the total α level. Scale and factor analyses were conducted to verify the scale’s reliability and construct validity. Confirmatory factor analysis of a half randomly selected sample was implemented by the Statistical Analysis System (SAS, Version 6.12). Path analysis was also implemented by SAS. Covariance Analysis of Linear Structural Equations (CALIS) was performed to examine the model. Furthermore, maximum likelihood estimation was used to appraise the parameters with the covariance matrix. The path model was modified for several times until the main indexes of goodness of fit implied the final model fit the data well. Generally, the α level was set at 0.05. Initial eigenvalue > 1 was the criterion in the factor analysis.

## Results

### Univariate analysis

Among 3222 respondents, 48.7% of them were male and 51.3% of them were female. The range of age was from 16 to 81 years and the average age was 33.8 ± 14.0. The majority (96.3%) of the respondents ware the Han nationality. As for the marriage status, the proportions of single, married and other status were 40.6%, 57.0%, and 2.4%, respectively. Occupations of the respondents distributed across a number of fields, such as worker (30.4%), service provider (26.5%), office worker (13.2%), farmer (5.0%), retired (3.3%) and others(including students, scientific and technical workers, teachers and doctors) (21.6%). Table 2 shows other social demographic characteristics.

**Table 2:** Mean scores of prior knowledge, health literacy, health behavior and health status by participant characteristic

**Participant characteristic**	**N**	**%**	**Prior knowledge**	**Health literacy**	**Health behavior**	**Health status**
			**Mean ± Std**	**Mean ± Std**	**Mean ± Std**	**Mean ± Std**
Age (years)						
16-22	882	27.8	7.26 ± 2.16§	22.51 ± 4.85§	14.81 ± 3.68§	9.82 ± 1.79§
23-29	684	21.6	8.08 ± 2.18†	22.65 ± 5.12§	15.35 ± 3.70§†	9.96 ± 1.80§
30-39	610	19.3	8.43 ± 2.22‡	21.74 ± 5.66§	15.78 ± 3.76§†	10.08 ± 1.79§†
40-49	502	15.8	7.80 ± 2.57†	19.04 ± 6.28†	15.53 ± 3.56§†	10.33 ± 1.78†
50-59	283	8.9	7.72 ± 2.48†	16.28 ± 6.46‡	15.35 ± 3.78†	10.37 ± 1.77†
60+	207	6.5	7.86 ± 2.60†	15.61 ± 5.91‡	15.45 ± 3.95†	10.36 ± 1.75†
*F* value			20.936***	119.911***	5.681***	8.442***
Education						
Less than middle school	206	6.5	6.29 ± 2.55§	14.32 ± 6.12§	14.00 ± 3.84§	10.04 ± 2.04
Middle school graduate	1016	31.9	7.20 ± 2.34†	18.21 ± 5.85†	15.01 ± 3.78†	10.14 ± 1.74
High school graduate	1277	40.1	7.80 ± 2.16‡	21.93 ± 5.28‡	15.30 ± 3.61†	10.03 ± 1.81
More than high school	683	21.5	9.27 ± 1.84#	24.51 ± 4.23#	16.25 ± 3.58‡	10.00 ± 1.82
*F* value			162.107***	311.262***	25.684***	0.980^NS^
Income(RMB yuan)						
<1000	327	10.4	7.01 ± 2.43§	17.36 ± 6.48§	15.26 ± 3.76	10.14 ± 1.83
1000-1999	1293	41.2	7.58 ± 2.18†	21.20 ± 5.73†	15.16 ± 3.67	9.98 ± 1.80
2000-2999	992	31.6	7.96 ± 2.39‡	20.82 ± 5.76†	15.40 ± 3.71	10.01 ± 1.87
3000-3999	338	10.8	8.80 ± 2.22#	22.58 ± 5.45‡	15.88 ± 3.63	10.15 ± 1.69
>4000	185	5.9	8.79 ± 2.21#	22.85 ± 5.74‡	15.46 ± 3.65	10.36 ± 1.66
*F* value			38.563***	43.721***	2.727*	2.366 ^NS^

*Note*: NS, no significant difference; *, *P* < 0.05; **, *P* < 0.01; ***, *P* < 0.001. The Student-Newman-Keuls method was used to control the total α level. There were significant differences between §, †, ‡ and # groups, but no significant differences within each group.

Table 2 also shows the differences among age groups, education levels and income levels on prior knowledge about infectious respiratory diseases, health literacy, health behavior and health status.

Prior knowledge score and health literacy score increased as education levels and income rose, but tended to decline with increasing age. Health behavior scores increased with higher levels of education and health status scores increase slightly with age. The effect of age on prior knowledge and health behavior had no linear trend.

### Correlation of variables

As seen in Table 3, the correlations between demographic characteristics and various scores reflected the same characteristic with Table 2. As for scores of prior knowledge, health literacy, health behavior, the correlations between each other were strong, while health status only has slightly strong correlation with health behavior.

**Table 3:** Correlation between demographic characteristics, prior knowledge, health literacy, health behavior and health status

**Variables**	**Age**	**Education**	**Income**	**Prior knowledge**	**Health literacy**	**Health behavior**	**Health status**
Age	1.000						
Education	-0.106**	1.000					
Income	0.032	0.324**	1.000				
Prior knowledge	0.051	0.349**	0.211**	1.000			
Health literacy	-0.386**	0.468**	0.173**	0.347**	1.000		
Health behavior	0.049	0.144**	0.044	0.321**	0.164**	1.000	
Health status	0.113	-0.023	0.032	0.008	-0.048	0.113**	1.000

*Note*: **P < 0.01.

### Path analysis

**Figure 2:**
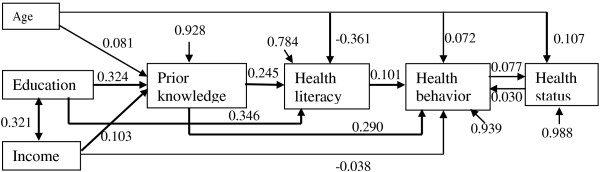
A validated health literacy model at an individual level with standardized coefficients.

Based on the proposed model on Figure 1, a path model was tested and validated, seen as Figure 2. The exogenous variables included age, education and income. The main indexes of goodness of fit implied the final model fit the data well. They are as follows: Fit criterion = 0.0034; Goodness of Fit Index (GFI) = 0.9990; GFI Adjusted for Degrees of Freedom (AGFI) = 0.9955; Root Mean Square Residual (RMR) = 0.0521; Chi-square = 10.2151, df = 6, *P* = 0.1159; Bentler's Comparative Fit Index = 0.9984. The proportion of variance explained by each variable in the model is: Prior knowledge: 13.8%, Health literacy: 38.6%, Health behavior: 11.7% and Health status: 2.3%.

Figure 2 shows the determinants of health literacy and its effect on health behavior, even the relationship between health behavior and health status. The bold arrows show the strong effects among variables, especially “Education”, “Prior knowledge”, “Health literacy” and “Health behavior”. Education is the most important factor. It strongly and directly affects both prior knowledge and health literacy. The higher the level of education, the higher one tends to score in terms of prior knowledge and health literacy. Prior knowledge is slightly affected by income, with those earning higher incomes possessing greater prior knowledge. Health literacy is also affected by prior knowledge and age; the effect from prior knowledge is positive and that from increasing age is negative. Health literacy is a direct influencing factor of health behavior, but its effect is weaker than that of prior knowledge. The strongest influence factor for health status is age. With increasing age, health status is better. Health behavior and health status have an interactional relationship, and the role of health behavior on health status is a little greater than that of health status on health behavior.

## Discussion

This study established and validated a health literacy model at the individual level. This model included socio-demographic characteristics, prior knowledge, health literacy, health behavior and health status. It is a simple empirical model rather than a complicated conceptual model.

### Socio-demographic characteristics as basic determinants

In the model, socio-demographic characteristics are the basic factors. In this research, a number of socio-demographic factors were tested, such as gender, ethnicity, marital status, and occupation. There was no significant difference between genders and ethnic groups when it came to measurements of health literacy. The main reason is that the awareness of the public on the prevention of infectious respiratory disease has been greatly increased with various intervention activities being conducted after the outbreak of Severe Acute Respiratory Syndromes (SARS) in 2003 and the outbreak of Highly Pathogenic Avian Influenza in 2006 in China. There was, however, a significant difference between the unmarried and married group, but the difference is explainable by age differences. In addition, there was a significant difference in health literacy across three categories of occupation. Highest health literacy scores were seen among students, scientific and technical workers, teachers and doctors. Office workers, service providers, general workers and other workers scored lower, while farmers and retired people scored lower still. Due to the strong relationship between education level and subsequent occupation, the effect of occupation on health literacy reflected the effect of education on health literacy in a similar fashion. Therefore, the model incorporated only three important factors: age, education and income.

Undoubtedly, educational background is the most important factor. In a structural equation, the coefficient of education background on health literacy was 2.35, which indicates that with each level of education (classified as primary school, junior high school, senior high school, college and graduate students), participants score almost 2.35 points more in the health literacy test, which is roughly equivalent to understanding eight percent more health information in daily life. This indicates how important education is for the promotion of health literacy. Education has the same strong effect on prior knowledge, and a further indirect effect on health literacy though prior knowledge. As an important social source of information, the effect of higher education levels on health literacy has been demonstrated in many studies [6,16]. In this study, we confirmed the quantitative relationship between education and health literacy, and the standardized coefficient (β)was 0.35 almost same with Cho’s study (β = 0.33) [16].

Age is the second important factor. Through careful measurement, we find that prior knowledge and health literacy tend to increase slightly among younger age groups, but then decrease significantly with age among the older age groups. Therefore, targeting the under-30 age group for the popularization and publicity of health literacy program – when perception and behavior form and develop stably – can promote their health skills and knowledge, bringing them lifetime benefits. For those aged over 30, health communication and health education must be consolidated due to the downward trend of knowledge and health literacy with aging. Conversely, the study found older age groups’ health status was better than that of the younger groups, with the 30–39 age group as the dividing point. This finding is contrary to what other studies have measured. The main reason for this is that the health status category was only concerned with the frequency an individual got a cold and the severity of such sicknesses because it is relatively easier and more feasible to measure the frequency of catching a cold and its severity than other kinds of infectious respiratory diseases. As we know, older people have often developed stronger resistance to these illnesses than younger people. For example, Kumar’s review of H1N1 flu shows that the virus is causing critical illnesses mostly in young adults. The researchers concluded that H1N1 (swine flu) primarily affects young adults who are in relatively good health and free of underlying illnesses [27].

Income is the weakest of the three influencing factors in this study. It has only a slight effect on prior knowledge. Usually, those of higher individual incomes own more sources of knowledge. Therefore, the measured negative effect of income on health behavior is an unexpected phenomenon, though the standardized coefficient is very little and the t value of -2.1416 is only just significant. Therefore, the relationship between income and prior health knowledge needs further research to confirm.

### Effects of prior knowledge

In this study, prior knowledge is defined as an individual’s knowledge at the time before reading, watching or listening to the health-related materials. Baker’s article cited the report of the Institute of Medicine’s expert panel, and gave a more expansive definition of health literacy which included conceptual knowledge as part of health literacy [20]. However, more researchers view conceptual knowledge or prior knowledge as a resource or a moderator that a person has, which facilitates health literacy, but does not in itself constitute health literacy [21-23,28]. This study finds that prior knowledge has a strong direct effect on health literacy. That is to say that a person with more health knowledge is better able to obtain, comprehend and use health information.

### Determinants of health behavior and health status

In the model, we confirmed that health literacy and prior knowledge are the top two determinants of health behavior. Prior knowledge’s effect on health behavior stands to reason, for example in the KAP model [29]. Health behavior and health status are interactional. In Baker’s model, health literacy is one of many factors that lead to the acquisition of new knowledge, more positive attitudes, greater self-efficacy, positive health behaviors, and better health outcomes [20]. In von Wagner’s model, health outcomes depend on a range of mediating processes, most obviously actions to promote health, prevent disease, or comply with diagnosis and treatment, which the author calls health actions [22]. In Paasche-Orlow and Wolf’s 2007 model, they proposed causal pathways between limited health literacy and health outcomes [21]. Their models distinguish three different types of health actions that mediate the impact of health literacy on health: access to and utilization of health care, patient–provider interaction, and self-care. In this study, health behavior mainly focused on self-care and utilization of health care, while health status reflected health outcome. However, health behavior and health status did not show a good relationship. The measurement of health status in this study was conditioned to respiratory infection due to the restriction of the project scope. It is obvious that respiratory infections are influenced by many things, not only individual behavior, but also a variety of biological and social factors. Therefore, the relationship between health behavior and health outcomes, and the effect of health literacy on health outcomes though health behavior need further study to validate.

## Conclusions

This model explains the determinants of health literacy and the associations between health literacy and health behaviors well. Education has positive, strong and direct effect on prior knowledge and health literacy. Health literacy is also affected by prior knowledge and age, the effect from prior knowledge is positive and that from age is negative. Health literacy is a direct influencing factor of health behavior. The most important factor of health status is age. Health behavior and health status have a positive interaction effect.

### Practice implication

In this study, we focus on a health literacy model at the individual level. We should also try to highlight the importance of future research to extend the scope of health literacy beyond the individual. The research indicates that medical knowledge and health literacy are the main determinants of health behavior and health status, so health educators and health care providers should focus on developing culturally sensitive educational materials using a variety of media. Increased staffing of health educators in clinical settings and community interventions would also help increase health literacy. We would like to develop an intervention that demonstrates how health literacy can be addressed to target community outcomes as opposed to individual outcomes. It is also important for this model to be tested — and likely revised — so that intervention strategies to mitigate the impact of low health literacy are informed and conceptually driven.

Limited by the project’s background, this study only measures health literacy where it concerns infectious respiratory diseases. Therefore, the feasibility of the model should be tested in regards to other diseases and aspects of health.

## Competing interests

We have no competing interests.

## Authors’ contributions

XS participated in the design of the study, performed the statistical analysis and involved in drafting the manuscript. YS participated in the fieldwork of the study and interpretation of data. QZ participated in the fieldwork of the study and analysis of data. YW participated in the fieldwork and involved in revising the manuscript. WD made contributions to design and participated in the fieldwork. NW conceived of the study and participated in its design. RX conceived of the study and participated in its design. CC conceived of the study, participated in its design and coordination. All authors read and approved the final manuscript.

## Pre-publication history

The pre-publication history for this paper can be accessed here:

http://www.biomedcentral.com/1471-2458/13/261/prepub
